# Early to Mid-Holocene land use transitions in South Asia: A new archaeological synthesis of potential human impacts

**DOI:** 10.1371/journal.pone.0313409

**Published:** 2025-02-12

**Authors:** J. Bates, K. D. Morrison, M. Madella, A. C. Hill, N. J. Whitehouse, T. Abro, P. Ajithprasad, K. Anupama, A. Casile, A. Chandio, S. Chatterjee, K. Gangopadhyay, E. Hammer, S. Haricharan, M. Hazarika, R. Korisettar, A. Kumar, C. Lancelotti, S. Pappu, O. Parque, C. A. Petrie, R. Premathilake, V. Selvakumar, S. Sen, M. Spate, M. Trivedi, G. M. Veesar, V. Vinayak

**Affiliations:** 1 Department of Archaeology and Art History, Seoul National University, Seoul, South Korea; 2 Department of Anthropology, University of Pennsylvania, Philadelphia, PA, United States of America; 3 CaSEs, Department of Humanities, Universitat Pompeu Fabra, Barcelona, Spain; 4 School of Geography, Archaeology and Environmental Studies, The University of the Witwatersrand, Johannesburg, South Africa; 5 ICREA, Barcelona, Spain; 6 Archaeology, School of Humanities, University of Glasgow, Glasgow, United Kingdom; 7 Museum of Archaeology and Anthopology, Department of Archaeology and Anthropology, Shah Abdul Latif University, Khairpur, Sindh, Pakistan; 8 Maharaja Sayajirao University of Baroda, Baroda, India (retired); 9 Laboratory of Palynology & Paleoecology, French Institute of Pondicherry, Pondicherry, India; 10 French Institute of Pondicherry, Pondicherry, India; 11 Laboratoire d’Ethnobiologie, IRD, Research Unit: PALOC (Patrimoines Locaux et Gouvernance) Muséum National d’Histoire Naturelle, Paris, France; 12 Department of Archaeology, Aror University of Art, Architecture, Design and Heritage, Sukkur, Sindh, Pakistan; 13 Department of History, School of Liberal Arts and Social Sciences, SRM University, Amaravati, Andhra Pradesh, India; 14 Department of Archaeology, University of Calcutta, Calcutta, India; 15 Near Eastern Languages and Civilizations Department, Price Lab for the Digital Humanities, University of Pennsylvania, Philadelphia, PA, United States of America; 16 Department of Humanities and Social Sciences Indian Institute of Technology Bombay Powai, Mumbai, India; 17 Department of Archaeology, Cotton University, Guwahati, Assam, India; 18 National Institute of Advanced Studies, IISc Campus, Bengaluru, India; 19 Sharma Centre for Heritage Education, Chennai, India; 20 SIAS, Krea University, Sri City, India; 21 Department of Archaeology, University of Cambridge, Cambridge, United Kingdom; 22 Postgraduate Institute of Archaeology, University of Kelaniya, Kelaniya, Sri Lanka; 23 Department of Maritime History and Marine Archaeology, Tamil University, Thanjavur, India; 24 Department of Archaeology, Jahangirnagar University, Savar, Bangladesh; 25 Department of Archaeology and History, La Trobe University, Melbourne, Australia; 26 Department of Anthropology and Stanford Archaeology Centre, Stanford, CA, United States of America; 27 Museum of Archaeology and Anthropolgy, Department of Archaeology, Shah Abdul Latif University, Khairpur, Pakistan (retired); 28 Indraprastha College for Women, University of Delhi, New Delhi, India; University of California Santa Cruz, UNITED STATES OF AMERICA

## Abstract

While it is clear that current human impact on the earth system is unprecedented in scope and scale, much less is known about the long-term histories of human land use and their effects on vegetation, carbon cycling, and other factors relevant to climate change. Current debates over the possible importance of human activities since the mid second millennium CE cannot be effectively resolved without evidence-based reconstructions of past land use and its consequences. The goal of the PAGES LandCover 6K working group is to reconstruct human land use and land cover over the past 12,000 years. In this paper, we present the first large-scale synthesis of archaeological evidence for human land use in South Asia at 12 and 6kya, a critical period for the transition to agriculture, arguably one of the land use transitions most consequential in terms of human impact on the Earth system. Perhaps the most important narrative we can pick out is that while there are some shifts in land use across these time windows, hunter-gatherer-fisher-foraging remained the dominant land use, and within this there was a mosaic of strategies exploiting diverse and complex landscapes and ecologies. This is not necessarily a new conclusion–it is not new to state that South Asia is comprised of many niches, but demonstrating the deep time history of how people have adapted to these and adapted them is an important step for modelling the impacts of human populations and thinking about their footprints in a longue-durée perspective. Despite the new development of food production between the early and mid-Holocene by overall area foraging life ways continued as the dominant land use practice into the 6kya time window. The development of agriculture and food production was not unimportant–it is the beginning of a land use that eventually comes to dominate the sub-continent, but at 6kya agriculture was restricted to specific contexts. Across 12kya to 6kya and different land uses, the use of mosaic ecologies, diverse strategies and the importance of water as a resource stand out as shared themes.

## 1. Introduction

It is clear that current human impact on the earth system is unprecedented in scope and scale [1–3], but much less is known about the long-term histories of human land use and their effects on biodiversity, vegetation change, carbon cycling, soil carbon dynamics, and other factors relevant to climate change. Debates about human impacts on climate focus on the period since the Industrial Revolution [4–8] and commercial agriculture including the large scale cultivation of crops like tobacco, coffee and tea and changing exploitation of forests as part of colonial expansions [9], as well as some discussion on prehistoric land use impact (e.g.: [7, 10]. Such debates have been rolled into wider discussions around the Anthropocene, its placement as geological epoch or event and whether this should be kept as a recent occurrence or pushed further back in time to account for longue durée human-environmental entanglements [4, 6, 11–13]. As part of such debates, it is important to recognize that long-term human impacts cannot be effectively resolved without multi-proxy evidence-based reconstructions of past land use and its consequences. Despite intense archaeological research over the twentieth century into many records of human action reliable data aggregation at supra-regional scales in many regions remains rare. Where data syntheses do exist, diverse research histories and procedures have resulted in uneven data coverage and quality, and inconsistent ways of classifying land use, making global-scale assessments a significant challenge [14]. In this paper, we present the first large-scale synthesis of archaeological evidence for human land use in South Asia at critical phases for transitional lifeways in the Holocene (12 and 6kya), including the transition to agriculture, arguably one of the land use transitions most consequential in terms of human impact on the Earth system [6]. South Asia has an extremely long history of human occupation, and is currently home to more than one billion people, making it a critical location for understanding the long-term histories and consequences of human land use.

**Fig 1:**
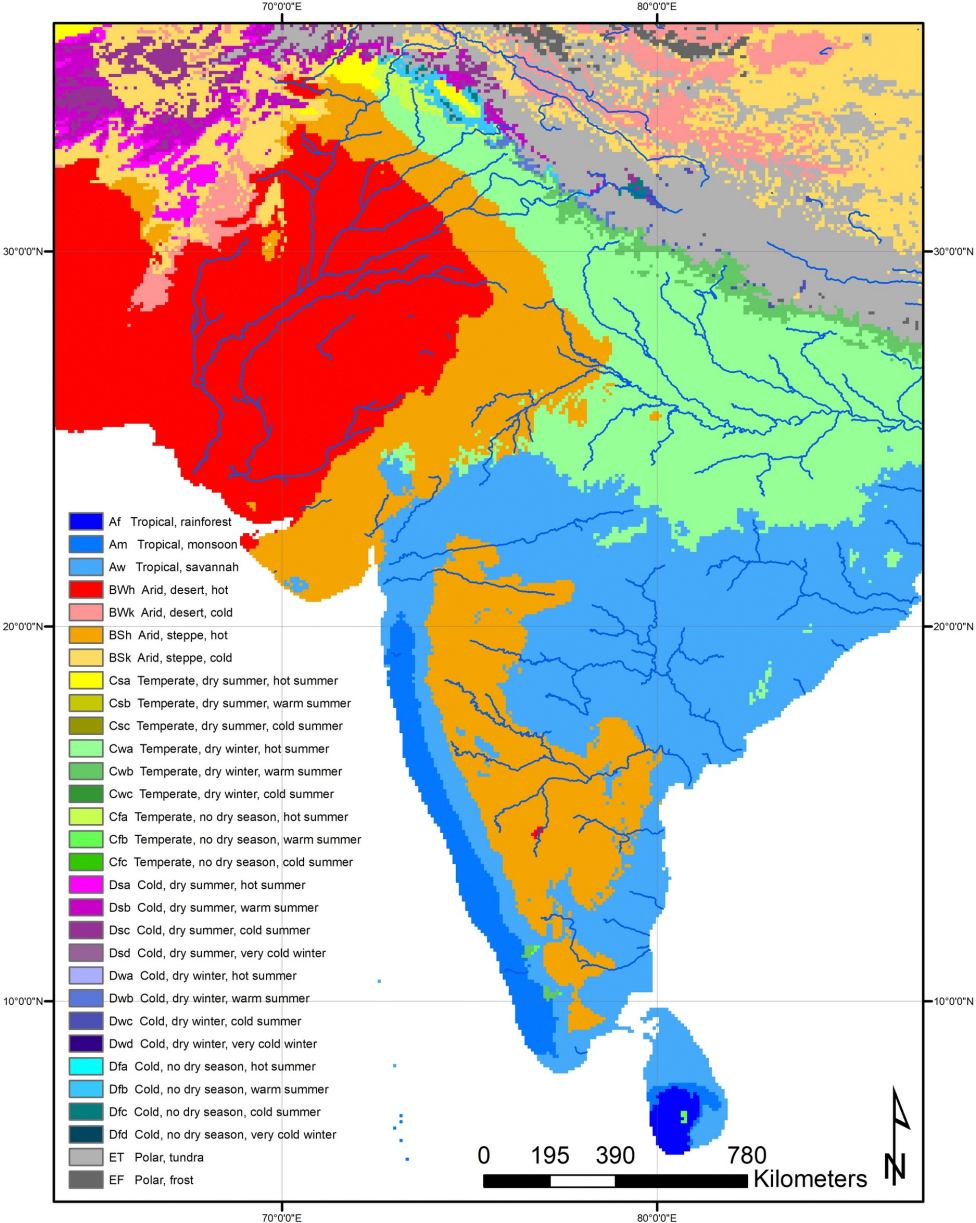
Environmental Köppen Geiger classes for South Asian subcontinent [20]. Figure created from data under a Creative Commons Public Domain Dedication CC BY 4.0: Beck, H. E. et al. Figshare https://doi.org/10.6084/m9.figshare.6396959 (2018).

Early farming in South Asia developed as a mosaic process, with multiple centers of plant and animal domestication as well as the adoption of already-existing domesticates [15–19]. Rather than the spread of agriculture from a single center, we see a diversity of strategies developing in different regions ranging from the limited use of domesticates by hunter-gatherer-fisher-foragers, to annual cropping and herding using diverse suites of cultigens, to more specialized modes of farming. Land use diversity continues to characterize South Asia today, where numerous forms of land use can be found including irrigated rice paddies, dry-farmed fields of wheat, millets, and pulses, agroforestry and shifting agriculture, mobile pastoralists, and hunting and gathering, to name just a few. In part, this high diversity of land use patterns follows from the significant environmental diversity of the subcontinent which ranges from tropical humid to temperate highlands and includes significant areas of aridity and semi-aridity [20] (Fig 1), but some may also relate to path-dependent histories of land use change. Here we set the foundation for a long-term analysis of land use change in South Asia by examining two time windows, one at12kya and the other shortly after at 6kya. This first-ever synthesis of archaeological data on land use for South Asia highlights the deep history of dynamic land use change as well as the presence of significant diversity within land use practices. While the latter may help elucidate the multiple transitions to farming in South Asia, data on the former are critical for assessing the potential environmental impacts of human activity on a subcontinental scale.

Documenting the history of land use is a necessary first step to assessing its environmental impact. Recent archaeological and paleoenvironmental research has challenged the view that hunting and gathering groups had little or no impact on vegetation and local environments, with landscapes such as the prairies of North America and the Australian Interior, among others, significantly affected by foraging groups using fire as a technology for enhancing edible biomass production [21]. While hunting and gathering thus cannot be discounted as drivers of landscape change, it is also the case that the transition to agriculture was one of the most significant transformations in human land use, leading ultimately to large-scale changes in vegetation, soil, slope, hydrology, climate and habitat as well as opportunities for new human social and political being. In South Asia, this critical transition took place in several locations between 12 and 6kya, each with identifiable local characteristics [15–18]. However, even after the advent of agriculture, pastoralism, hunting and gathering continued to be practiced until the present day across South Asia [22–24], highlighting the diversity of food acquisition and land use strategies across the subcontinent.

The analysis of South Asian land use presented in this paper has been part of a larger effort to commensurate and aggregate archaeological evidence of past human land use at a global scale through the Past Global Changes (PAGES) LandCover6k (LC6k) working group [3, 14, 25]. Through the use of a unified land use classification and land use variables, as well as a consistent data structure [14] this paper presents a large scale synthesis and analysis of human land use across all of South Asia in these two important time windows for land use and changes in lifeway, the Early Holocene (12kya) and the Mid Holocene (6kya). By including the entire subcontinent and all forms of land use within it, this analysis highlights both the mosaic nature of early agriculture and the long-term persistence of hunting and gathering, differing from accounts which begin with the oldest example of agriculture in South Asia (which falls between these two time windows, as discussed below) and trace the history of farming as a singular narrative. Our approach places agricultural land use into a larger context and allows for quantitative assessment of its significance. The analysis presented here provides novel insight into shifts in land use that occurred across this critical period, representing a significant advance over approaches based either on spatially coarse data [26] or population-based models [27–29].

## 2. Chronological challenges: The problem with periods and creating a unified understanding of South Asian land use in deep time

The PAGES LC6k working group consisted of an international working group comprising archaeologists, historians, geographers, paleoecologists and modelers building a global database of land use and land cover changes over the last 12,000 years. The LC6k land use subgroup has been working to integrate the vast amount of fragmentary and disparate data produced by archaeologists and historians into a single global database that can be used to improve earth systems models [3, 14]. Using a unified land use classification system, as well as a consistent data structure [14], a significant first step in building this database was the construction of continental-scale data sets that represent the building blocks of a global assessment.

Through this approach, the LC6k team has endeavored to provide a foundation for understanding where, when, and how humans transformed specific environments. Although the project has aimed to include several later time windows, 12kya and 6kya were set as our initial goal, based on conversations amongst the archaeologists, palynologists and climate modelers [3]. However, the choice of 12kya and 6kya poses a unique challenge for South Asian archaeology.

Terminology used to describe material from 12 and 6kya can be highly variable, sometimes referring not so much to absolute dates or even periods of time, but rather to cultural categories such as Mesolithic or Neolithic, which are not temporally uniform across South Asia [30, 31]. Terms such as Paleolithic, Mesolithic, and Neolithic were originally borrowed from European archaeology [32] without consideration of fit and appropriateness for the unique lifeways in South Asia [33–36]. Extensive debate over terminology has occurred within South Asian archaeology and there has been a general recognition that these terms should be replaced with more regionally specific terms (e.g. [37–39].

The shortage of radiometric dates means that many chronological assignments must be based on stone tool typologies [40], in which microliths are a critical type. However, microliths are an especially complex tool type for use in chronologically-linked typologies. Microliths appear to have been made and used over a very long time period which both pre- and post-dates the time windows discussed here. Indeed, microliths are noted by several authors [33, 36, 41–43] as stretching chronologically from 48-45kya to the early centuries CE. Yet as Roberts et al. [36] note, a wide array of ‘ages’ or ‘periods’ including Mesolithic, Meso-Neolithic, Bandarawelian, Indian Late Stone Age, and Microlithic have been linked explicitly to the ‘microlithic tradition’ and the presence of microliths used as markers of these ages. Microliths continue to be used as chronological indicators, but the longevity of microlith use poses certain risks in doing so. This is especially important here as microliths are, with good reason, often seen as a marker of the Mesolithic; unfortunately, their continued use means that sites noted to be Mesolithic but without any other chronological evidence must be carefully scrutinized. Indeed, if microliths alone defined the Mesolithic then the latter could be argued to extend from 48-45kya into the post CE centuries [43, 44]. For this reason, we have been cautious in our assessment of site chronology.

Some recent work has reinforced the view of microliths as markers not of chronology but as markers of developing technologies and social complexity [36] that played an important part in broader Pleistocene toolkits, continuing in use into the Late Holocene. Microliths in this view can be seen not so much as specific to a chronological period but as a technology that cuts across traditional periodizations [36]. Wedage et al. [43, 45] suggest that regional variations on the theme of lithic miniaturization are reflections of a diverse toolkits for diverse environmental adaptations [46], rather than reflections of a cultural horizon, as traditional typologies would have it. The most recent migration of *Homo sapiens*, possibly around 60kya, was facilitated by flexibility in approaches to food procurement, as was human colonization of the whole of the subcontinent throughout the Late Pleistocene and into the Holocene. Flexible microlithic technologies remained important into the 12k and 6k time windows this paper explores.

Given this background, when assessing data for this paper we included sites in the database only when clear chronological assessments were provided; for example, if authors included a specific chronological period or if radiocarbon dates were reported. Where chronological and/or land use data were considered problematic, this has been noted in the data quality section, as outlined below. We have also tried to be explicit where we have ‘no data’, to illustrate gaps in knowledge and separate the lack of data from the lack of past human land use (see Fig 7). This cautious approach reduced the number of sites significantly. At present, South Asia does not have a public database of radiocarbon data.

## 3. Methods and materials

### 3.1 The LandCover6k land use database and data collection

The PAGES LC6k working group was a global collaborative endeavor involving researchers from different subjects working across the world and across the Holocene. As such, the specific goals of the PAGES LC6k working group required a purpose-built classification that not only separates cultural activities from possible outcomes, but also be used globally and across the entire Holocene. Because land use and landcover are not always linked in a consistent way–more than one form of land use can result in similar land cover, for example [14]–we based our land use classification on the ‘uses’ people made of the land, as evident from archaeological and historical data, including plant and animal remains, rather than on paleovegetation data, which arguably track the effects of land use, alongside non-anthropogenic factors. The PAGES LC6k working group goals included understanding land use-land cover interactions; the land cover track of the project used aggregated pollen data to address these changes (see [3, 14] for more discussion).

**Fig 2:**
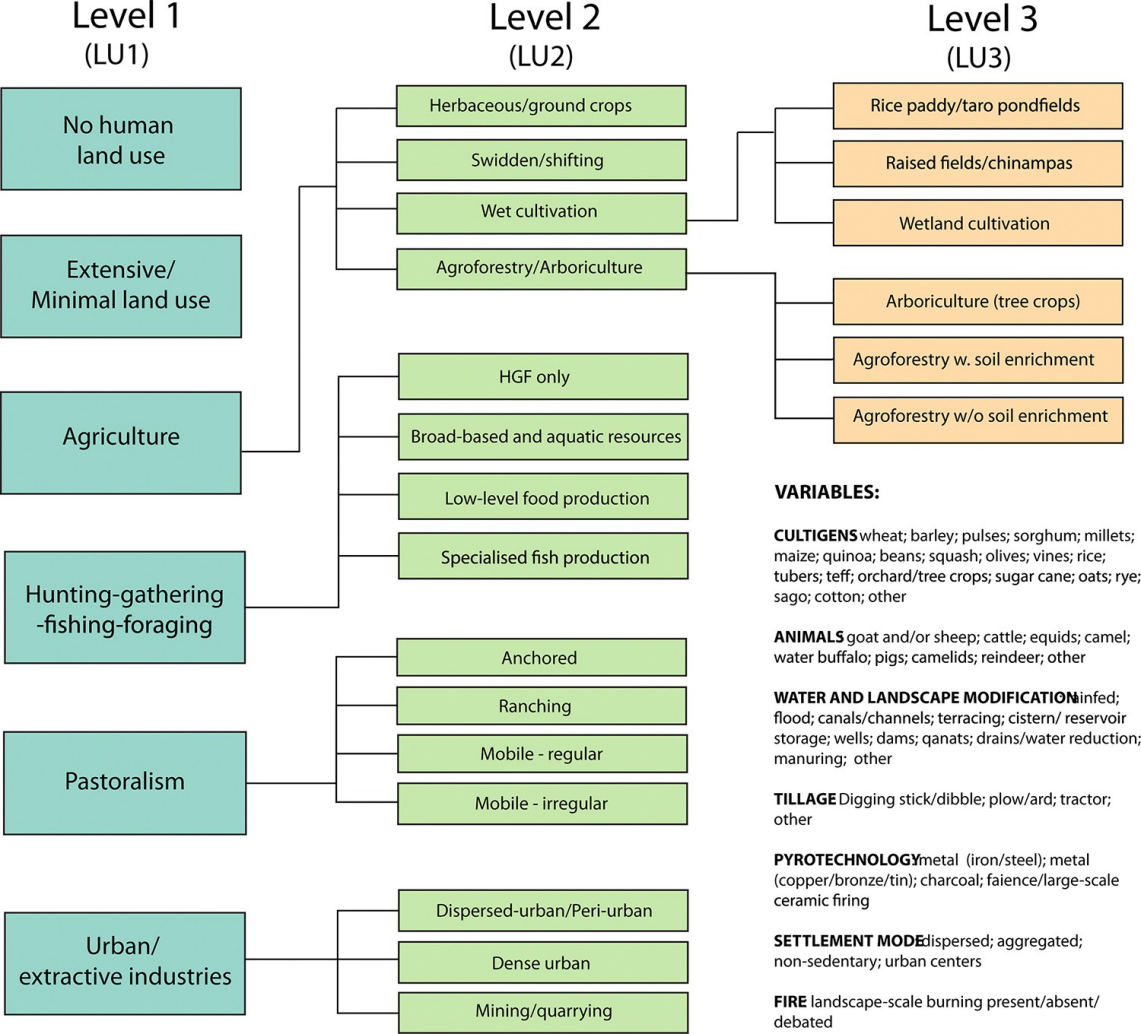
Nested hierarchies of the PAGES LandCover6k land use classification system. (reproduced from [14]: Fig 1). Reprinted from (14) under a CC BY 4.0 license, with permission from PlosONE, original copyright 2021.

The land use database and classification system is a three-level hierarchical classification ranging from the most general land use levels (LU1), including broad categories like ‘agriculture’ and ‘hunting-gathering-fishing-foraging,’ (HGFF) to more specific land use levels (LU2 and 3), meant to provide finer-grained details on land use [14] (Fig 2). The database includes information on domestic crops, animals, irrigation, tillage, pyrotechnology, settlement mode (aggregated vs. dispersed), and regional-scale burning. Data coverage and data quality are also recorded in the database; these are critical issues in South Asia where archaeological data, and especially the analysis and reporting of faunal and floral data, can be patchy and of uneven quality. Morrison et al. [14]: SI) contains more information on the database, including an overview discussion of data coverage and quality as well as an expanded discussion of chronological issues.

The database is constructed as a custom geodatabase in Esri’s ArcGIS platform. This geodatabase stores data in an 8km x 8km global grid. This grid size is a compromise, meant to replicate the resolution of data used by the rest of the PAGES LC6k community [47] even if it is coarser-grained than archaeologists often envision. The land use values recorded for each cell are meant to represent the dominant land use class for each 8x8 km grid cell (64 km^2^ area); interpretations about land cover made on this basis will need to specifically define the percent of land within each cell under use. What land use implies specifically for vegetation change is yet another question–regions used for HGFF and subject to periodic burning, for example, might have a different vegetation signature than a comparable region under HGFF that is not subject to anthropogenic burning. Thus, neither land use maps nor population maps can be unambiguously transformed into vegetation maps although both human population and human land use are potential drivers of vegetation change. The PAGES LC6k project has worked in time slices (as termed in [14] or time windows as we prefer here, focusing on periods that are of interest both to the climate modellers and to archaeologists. These time windows have a +/- error of 250 years either side of the boundary. Thus when we discuss in the paper data from 12kya or 6kya we mean data linked to the 500 year window around the specific moment (12,250–11,750 years ago and 6250–5750 years ago). The SI contains the 12kya and 6kya database for South Asia (dx.doi.org/10.6084/m9.figshare.6025748) as well as tables of the sites used in the analysis.

The data was collected through a series of workshops undertaken by the authors of the paper who are experts in the time periods and regions of study. Formal workshops were held in Delhi and Pondicherry in 2019, followed by smaller group meetings in Philadelphia and online. The workshops led to the construction of paper maps and a database. The paper maps informed rules in combination with decision making explained below, that were in turn used to populate the LC6k digital database for South Asia. The figures shown in this paper are the visualizations of this LC6k database and mapping exercises. Throughout the text the individual decisions and rules underlying these are explained.

When interpreting the maps that follow, it is important to keep in mind what these maps represent, and how they fit into the larger goals of the LC6k project. LC6k was designed to produce continuous, composite land use maps for the entire globe for each time slice.

To do this we have had strike a balance between the scale of data that archaeologists are comfortable with, generally smaller/local scales, and the scale of data needed for this research to be useful outside of archaeology: a larger/global scale. This created a fundamental tension between the need to fill in every 8x8km square in the entire globe, and the limits of available data and human knowledge. Archaeological data is patchy even in the most densely studied areas, but the LC6k database does not allow “No Data” entries. Instead we have utilized the best data available to come up with the land use type that was probably the most common (but necessarily the only) land use for each and every grid cell. Each regional working group approached this problem in different ways, depending on the available data. In the case of South Asia we have brought together much unsystematized data (in the form of expert knowledge) with the limited site data (from both published and unpublished information regarding the chronology of the site and the land uses present), interpolated and mapped using rules regarding broader cultural and environmental (ruggedness, elevation, aridity etc.) data. This has not always been optimal, but the variability in the data is clearly reflected in the quality and coverage data (see 4.4 below) and even with the level of interpolation needed to fill in this map, such mapping and database work still improves on the resolution and detail in the most commonly used land use models.

### 3.2 Land use categories used in this analysis

Of the potential land use categories in Morrison et al. [14], there are four LU1 categories relevant to the 12k/6k South Asia analysis. The first is no land use, used for regions without any human presence, narrowly defined as locations for which there is a demonstrable lack of human inhabitation. This assessment can be difficult to make, but was used here for places where a lack of archaeological data coincides with contexts such as isolated islands, extremely high and rugged mountains, or land that was underwater at the time. The category of minimal/extensive land use refers to areas where human land use was scattered and transitory, where people may have occasionally passed through, camped, foraged, or conducted other ephemeral activities. If there were known regions of human settlement separated by areas without evidence of land use, we assume that people had at least passed through those regions.

The most widespread form of land use in South Asia up to the mid-Holocene is expected to be LU1 hunting-gathering-fishing-foraging (HGFF), as defined in Morrison et al. [14]: SI). Within this broad category, locations with sufficient evidence can be sub-classified as LU2 hunting-gathering-foraging only, broad-based and/or marine/aquatic resources, low-level food production, and specialized fish production. The guidelines for assigning a HGFF designation note, “Many historically and ethnographically documented hunting and gathering groups practice some form of management of wild plants and/or animals, altering landscapes and creating what Lee [48] refers to as “engineered niches.” Since the line between exploitation and production of a resource can be ambiguous, the existence of some level of resource management should not disqualify an area from being coded as HGFF.

The LU2 sub-classification low-level food production (LLFP) is nested within HGFF in recognition of the gradations in forms of resource use. As noted by Morrison et al. [14]: SI) in the definition of this term, it is not strictly necessary to the database in that the presence of domesticates is coded separately. Sites like Langhnaj, where foragers also had access to domesticates [49] would still be considered HGFF.

Agriculture is of interest to the 6kya time window in particular, as it is around this period we see debates about the arrival of Near Eastern and African crops and the use of rice in South Asia [15–19]. LU1 agriculture has several LU2 sub-classifications; herbaceous ground crops, swidden/shifting cultivation, agroforestry, and wet cultivation. These are critical distinctions for land cover and for assessing human impact in general, but for the time periods covered here, there are very few instances where LU2 classifications can be made on the basis of available data. This is important for comparison with other land use syntheses, as discussed below. While it may be tempting to extrapolate back in time from later forms of land use–for example, to assume that a region where shifting cultivation was practiced in later periods was also under shifting cultivation earlier–such assumptions are not warranted without supporting evidence (cf. [50]).

With these potential LU classifications and sub-classifications in mind, it is therefore to the data synthesis we now turn.

## 4. Data synthesis

### 4.1 South Asia before 12kya

The South Asian subcontinent was a pivotal location for hominin migrations since the Early Pleistocene [45, 51–55], sitting at a key geographic and biogeographic boundary between the Sahara-Arabian/Afrotropical ecologies of the African continent and the Middle East and the Palearctic and Sino-Japanese ecologies of South East Asia and Australasia [45, 52].

A great deal of research has been done into the period of early human colonization into South Asia (e.g. [36, 45, 52]. Microlithic tool technologies were developed and widely used by hunting and gathering groups [33, 36, 43, 45, 56–58]. Alongside microlithic technologies seen from as early as 45kya, other forms of material culture from the Paleolithic include beads, bone and antler tools. The development of diverse burial practices [30, 33, 59, 60] indexes early socio-cultural variability, while population size increased over time, potentially linked with the stability afforded by MIS (Marine Isotope Stage) 3 [43, 61].

**Fig 3:**
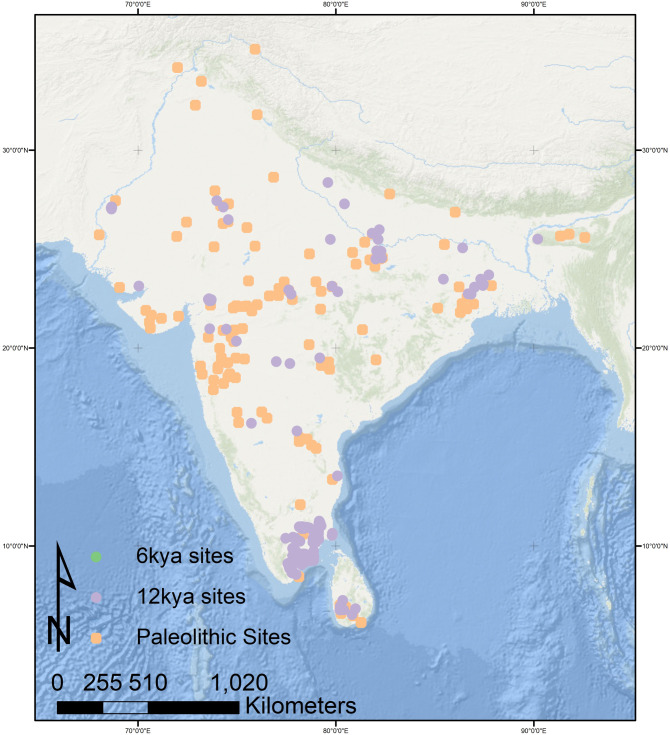
Comparison of Paleolithic sites (45-20kya), with 12k and 6k time windows. There are notably fewer sites in both the 6k and 12k time windows than in the pre-20k time period, with particular clusters in 12k and 6k that are discussed below. Created by authors using base maps from Esri. Base map image(s) is the intellectual property of Esri and is used herein under license. Copyright © 2020 Esri and its licensors. All rights reserved. Sources for base maps: Esri, GEBCO, NOAA, National Geographic, DeLorme, HERE, Geonames.org, and other contributors.

In South Asia across the Late Pleistocene period from 45kya we see several sites (Fig 3), each with their own variation on the microlithic technological suite. Sites like Fa Hien and others of similar date in Sri Lanka show a diversity in microlithic technological strategies adapted to both the raw material constraints and the rainforest environment [45] cf. [36, 42, 45]. Occupants of Jwalapuram 9 in southern India used a microblade technology to exploit the resources found at the juncture of the forested upland and lowland river valley, where the site is located [62]. This is also seen at other sites: microblades and flakes seen at Mektakheri and Patne in the Narmada Valley [30, 41]; blades and flakes at Dhaba 3 in the Middle Son Valley, with a gradual introduction of backing over time [56]; and a more complex assemblage of geometric microliths, backing, and core reduction for microblades, microblade cores and backed artifacts at Buddha Pushkar on the edge of the Thar Desert [46].

Paradoxically, the history of South Asia’s deeper past is somewhat clearer than that of the Early Holocene at 12kya, in part because of the chronological and typological issues outlined above. With few radiometric dates, microliths being used into the Late Holocene and other secure artifactual markers for chronology such as ceramics absent, the map of reasonably well-dated archaeological sites at 12kya is much sparser than that for 45-20kya (Fig 3). This difference probably does not indicate population loss or some other cultural change, but rather stems primarily from problems of systematics, a shortage of radiometric dates and likely due to the ephemerality of sites. There are many thousands of sites documented in the literature (e.g. [63] said to date to the Early Holocene; unfortunately, while most contain microliths, few have other chronological indicators. We were forced to exclude these sites from our analysis, but acknowledge that at least some of them were likely occupied 12kya.

### 4.2 South Asia at 12kya (time window 12,250–11,750 years ago)

At 12,000 years ago, the environment was facing a change. As the Earth moved out of the Last Glacial Maximum, glaciers retreated and conditions stabilized into warmer and more moist conditions. At the start of the Holocene the Younger Dryas event caused a brief return to the colder and more arid conditions, but following MIS (Marine Isotope Stage) 1 (starting date 11,700 years ago) the Holocene period of warmer and regionally variable climate records was established. While the Younger Dryas has a good record in other regions of the world, particularly the Near East, in South Asia the picture if more complex, in part due to the lack of or patchy nature of directly geographically correlated palaeoclimate records for the region. However, it can be noted that in the Ganges plains, for instance, there is are several records of fluctuations between warm, wet and arid periods between 15,000–10,000 BP [64–66]. Following this the return to warm, wet conditions is seen. It can be noted that while relatively stable, there have been climatic fluctuations throughout the Holocene such as the 8.2kya and 4.2k event [64–67]. Overall, the Holocene has been a period of generally milder, warmer and more stable climate than the preceding Pleistocene. Sea levels rose with the glacial retreat [68–70], though this had variable impacts along South Asian coastlines and modern sea level was not established until 3-4kya, and the monsoon system–critical to South Asian ecologies–faced some fluctuations (e.g. [71]. The 12kya time window therefore represents a point of change towards warmer conditions, with some cooler and arid events at the start.

**Fig 4:**
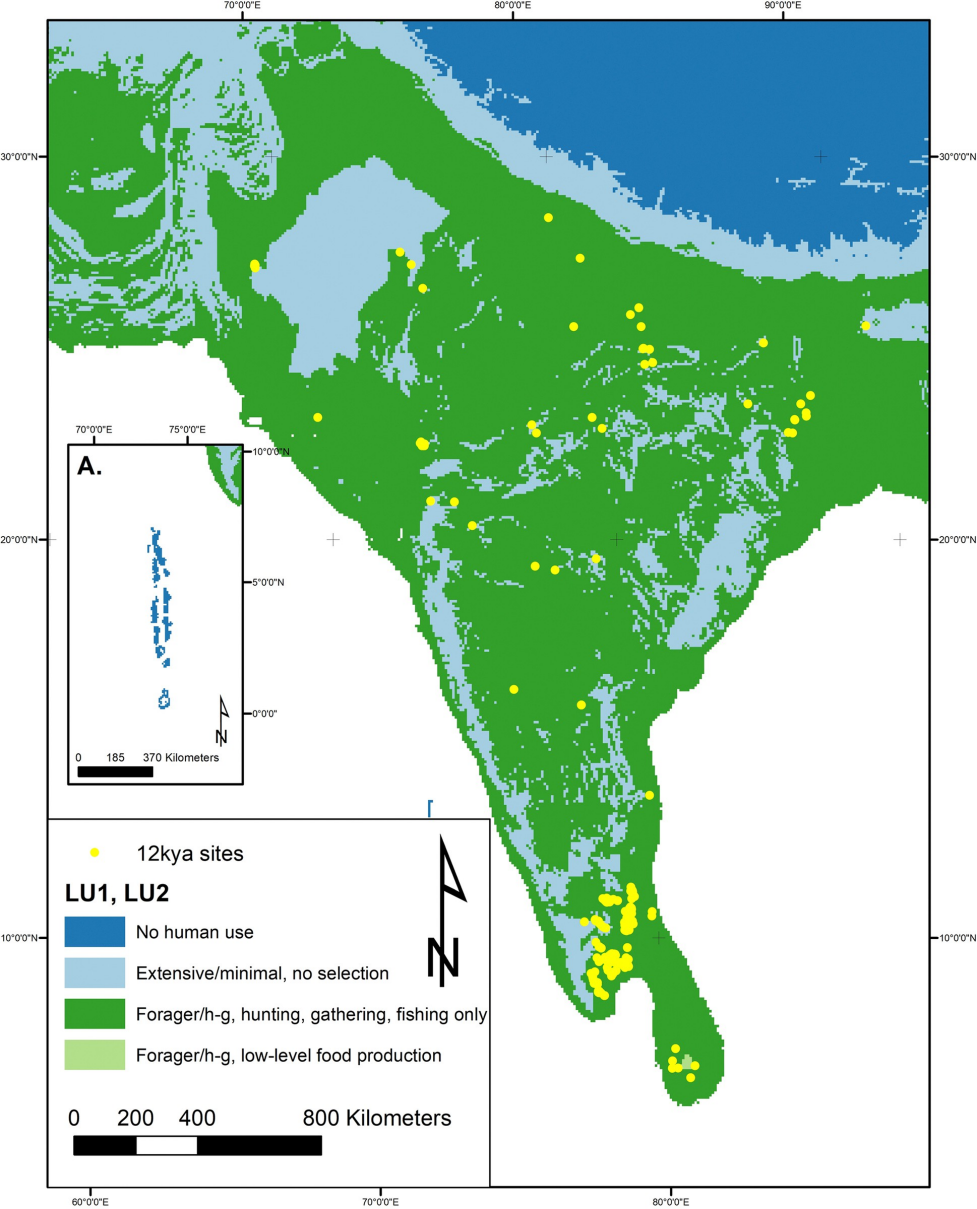
Land use map at 12kya (12,250–11,750 years ago). There is a notable spread of LU1 HHGF across the majority of the subcontinent, with patches of minimal-extensive and small points of LU2 LLFP. Note the difference in sea-level between 12k and the modern coastline creating different potential land to use (e.g.: Sri Lanka-mainland land bridge). Inset A shows Maldives, which are included at 12k due to the lower sea levels creating a greater coastline. As will be apparent by 6k, when sea levels rise to reach modern levels, such land masses no longer provide enough area to merit their inclusion within the land mass grid. At 12k though they form a large enough landmass due to lower sea levels, but present no evidence for human land use. Map made by authors using ArcGIS and LC6k database.

At 12kya South Asia, human land use consisted of some form of hunting-gathering-fishing-foraging (HGFF) or what we have termed minimal-extensive movements (use but not occupation) through the land. It is highly likely that there were significant variations in land use strategies within the broad HGFF classification, but at present few studies provide data appropriate for a detailed understanding of mobility, seasonality, or landscape modification. This will be an important direction for future research but at present we can gain some clues from the associations between archaeological sites and specific bioregions. In general, we presume that Early Holocene residents of South Asia were mobile and that unless we have good reason to think that humans were entirely absent from a specific region, much of the land in between known archaeological sites would have been at least occasionally used and/or traversed, giving it a classification of minimal/extensive land use. Current data do not allow more specific sub-categories of level 1 land use to be designated, so all areas under regular human land use are simply identified as HGFF (LU1). From this broad overview we can break down some more specific regional/thematic patterns within the 12kya data (Fig 4).

#### 4.2.1 Higher elevation settings

While higher-elevation areas across the region do not appear to have been inhabited at 12kya, it is probable that people passed over and through these areas, leading to their classification as minimal/extensive land use (see [72]. For all areas, grid squares with a mean elevation above 4,000m (all elevations as asl) were coded as no human land use. Elevations below 4000m but above 2000m were coded as minimal extensive. Upper Paleolithic HGFF sites in the Himalayas have been found at 2000m in Southern Tajikistan, suggesting some land use at this altitude [73, 74], though these sites were likely utilized only seasonally. Sites above such altitudes are more ephemeral, and exploitation of the environment is likely best characterized as minimal/extensive [75]. Meyer et al. [76] have argued that foraging may have occurred at higher altitudes (above 2000m) by citing Chusang, a foraging site at 4270m dating to c.12,700 BP, but the dates of this site are disputed (alternative dates suggest occupation at 8500 BP) and the nature of the occupation is also questioned. Beyond Chusang, all other known sites are represented by surface or ephemeral finds only (d’Alpoim Guedes pers. comm.), suggesting less intensive forms of land use.

The higher elevation areas of densely forested regions in Bhutan, Northeast India, and Bangladesh pose specific challenges. Difficulty of access and a lack of research [77] may be partially driving the sense of these regions as very lightly occupied in the Early Holocene, but it is difficult to be certain. The Himalaya elevation rule has been extended to this area, but the challenges inherent in this are recognized, and are noted in the data coverage and quality below. However, in peninsular India, based on consensus understanding of human occupation of the Western and Eastern Ghats at 12kya, simple elevation rules cannot adequately define LU boundaries and more complex rules are required. Land use categorization in these regions was accomplished by creating a Terrain Ruggedness Index (TRI) for all of India and then choosing an arbitrary cutoff value that adequately captured the western and eastern Ghats. All grid squares where the mean ruggedness is above this threshold, except grid squares that contained a known site, were coded as LU1 minimal-extensive.

#### 4.2.2 Low and mid-elevation settings

Beyond places like Chusang, most documented sites from this period are found in lower or mid-elevation settings. Sites such as Budha Pushkar [46] on the Thar Desert margins, Sarai Nahar Rai [39, 78], Chopani Mando [78, 79], Mahadaha [78, 79] and the Belan Valley Area sites [78] in the Ganges plains, Patne [30] in Maharashtra, and Batadomba Lena [57] in Sri Lanka all provide examples of locations of LU1 hunting-gathering-fishing-foraging land use. In the 12kya time window, there are potentially more than 100 known sites in the Belan Valley [80, 81] and c.100 sites in the Son Valley [80] though this relies on earlier definitions of what constitutes the Indian Upper Paleolithic and caution around their dating is needed. In the middle Ganges Valley, 204 identified sites were identified by Pal [81]: 177 sites in Pratapgarh district as compared to 6 in Allahabad, 5 in Sultanpur, 14 in Jaunpur and only 2 in Varanasi district. This distribution is somewhat patchy and ever changing due to continuing work, and likely the result of uneven exploration practices [81], but it does illustrate that HGFF land use systems were well established by 12kya in low-mid elevation regions.

Importantly, these areas were environmentally diverse, ranging from semi-tropical to semi-arid and exhibiting a range of available resources. Foraging strategies were thus also likely to have been diverse; documenting these strategies remains an important area for future research. While most excavated contexts lack faunal material, places where these are preserved and have been studied such as Jwalapuram Locality 9 in Andhra Pradesh [33] provide important insights into local foraging strategies. As more data of this sort become available, it may be possible to refine our analysis of HGFF land use across South Asia.

The Thar Desert is an exception to the widespread classification of low-lying areas. The Thar Desert is a region of prolonged high aridity, and presently extends 300,000 km2 through the Indian states of Rajasthan and Gujarat and the Pakistan province of Sindh. It is bounded by several significant geographic, climatic and ecological features. To the west there is a sharp increase in relief due to the subduction of the Indian tectonic plate under the Eurasian plate, while in the east the Aravalli Range creates a marked boundary.

The eastern border of the Thar is marked by a longitudinal gradient of the Indian Summer Monsoon. Paleoenvironmental data (see for example [82–87] suggest that this region has undergone significant climatic variation since the pre-Holocene, with the margins of the desert extending and contracting. These changes are tied to the dual rainfall systems (Indian Summer Monsoon and western/winter rainfall) that have complex multi-millennial, centennial and decadal systems (e.g. [88–94]. By MIS (Marine Isotope Stage) 2, although dune formation was well beyond modern arid zone limits, the margins of the desert were critical to human exploitation and paleoenvironmental adaptation [46]. The Thar may have been re-colonized with the resumption of more humid conditions in the Holocene, but clear and continuous human occupation has yet to be found in the core area for either the 12k or 6kya time windows, and as such we have coded it as minimal/extensive land use using the modern boundaries to define it. While there may be sites within the Thar around the playas that were being used for HGFF, currently the data shows only scatters of lithics and little additional analysis [95], and as such currently we are only able to infer that people were moving through the region rather than using it in a more intensive fashion.

From the pre-Holocene, we see examples of sites within the Pushkar valley in the Aravallis that suggest a route of migration and communication between the west and central India [46]. This would therefore suggest that the Thar was at least a route for movement. At the same time, the western edge of the desert, in what is today the province of Sindh, has evidence (albeit all from surface finds) of frequentation by HGFF groups with an Indian Middle Stone Age and Late Stone Age (microlithic) technology [96, 97].

#### 4.2.3 Coastlines and river deltas

The coastal regions of the subcontinent represent a different eco-zone for exploitation, with possible access to marine as well as terrestrial resources. The question of sea level change is important for modeling land use, not just along the coastlines of South Asia, but also in the many river deltas of the subcontinent. The coastline of the entire region was very different at the 12kya time window compared with the present. Modern sea levels were likely reached by 3400 BP, though highs could have been achieved at c.3000 BP before some rebalance occurred. In Sri Lanka, the lower sea level of the Early Holocene [98] would have exposed a land bridge to the South Asian subcontinent from the Last Glacial Maximum (LGM) until rising sea-levels led to the development of its island status after the Early Holocene [36, 99, 100]. For the initial time window of 12kya in this paper, the land bridge to Sri Lanka is therefore modelled as still present and would have filled in an additional 462 8x8km grid squares and so has been included in the maps and coded as HGFF reflecting other sites.

Boivin and Fuller [101] note that sea level change may also have been stark in regions where inland rivers drain, such as along the Makran coast and the Indus delta regions where sedimentation and sea level rise may have buried sites. Post-Pleistocene glacial melt caused coastline changes in the Early to Mid-Holocene due to sea level rising [102]. This would have had particular consequences in the Persian Gulf and around the Indus delta, for example, due to its shallow nature [102]. Rising sea levels starting in the Early and culminating in the Mid-Holocene may account for the lack of 12kya shell midden finds in the Indus delta and related areas as these may now be under water [101]. However the presence of shell middens in other areas with different coastline dynamics in the 6kya (see below) suggests we should find evidence of their earlier histories and other site types in now submerged areas, but without more detailed study in the region (see discussion in 91), this will remain speculative. There is only partial evidence of where the coastline changes would have been in these regions, and so we have not added a reconstruction of altered coastlines on the maps so as not to stretch the bounds of our interpretations.

Submersion is not the only issue–erosion and sedimentation are key points to contend with. Again, taking the Indus delta as an example, there are no sites reported on the delta coastal plains [101], and sites dating to the terminal Pleistocene and Early Holocene may now be eroded away or deeply buried [103]. Similar challenges are faced for parts of the main Indus alluvial plain and in Sri Lanka [36, 98]. Data quality may therefore be impacted in river delta regions for early time periods, but also in later times due to river shift or sea level fall [104, 105].

Different challenges are faced in the Bay of Bengal, on the Ganga-Brahmaputra-Meghna flood and delta plain. The sediments seen today formed during the Holocene [106, 107] and accumulated and/or were eroded according to multiple factors including the active river channels, the intensity of the summer monsoon and variable sea levels throughout the Holocene [106, 107]. The current coastline is likely an artifact not only of the environmental conditions of only the last few centuries but also of British intervention [108] and then more significantly in the 1960s when dams and other systems were put in place to control sediment flow and build up as a response to port access issues [107]. Based on Heroy et al. [109] and Mukherjee et al. [106], the coastline at 6kya can be placed further inland than it is today, and at 12k even further inland, a region now buried under deep layers of sediment.

Though the western upland areas of the Bengal basin have evidence of HGFF land use from 12kya at sites like Birbhanpur [110], Paruldanga [110–112] and Midnapur [110, 111], there is no evidence for land use in the Bengal Basin proper until 4000 BP [107, 110, 111]. Within what is now Bangladesh, no well-dated archaeological sites are found for either the 12kya or 6kya time window. This is reflected also in the Subarnarekha Delta to the southwest where there is evidence for HGFF in the mid basin but no human land use on the coasts until after 3kya [113, 114]. However, given that there is uplands evidence for human land use, we extend this down to the basin and apply HGFF.

A cautious approach to coastlines and river details has therefore been used, with a recognition that data quality is likely to have been severely affected by numerous issues of changing levels, erosion and burial.

#### 4.2.4 Islands

A diversity of island contexts are found within the bounds of South Asia, including the outlying Maldives, Lakshadweep, Andaman, and Nicobar island archipelagos, the latter three Union Territories of India. The Andaman and Nicobar Islands in particular have been argued to have very long occupational histories [115], but this is at present not supported by archaeological evidence (cf. [116]. As such, we have provisionally coded all these island areas as having no human land use [117].

This stands in contrast with Sri Lanka. Sri Lanka lies 48 km off the southern tip of the South Asian subcontinent, on the same continental shelf. Sea level rise and fall will have affected Sri Lanka’s island status. At 12kya, Sri Lanka was connected to the South Asian mainland by a land bridge [36, 98–100], but as sea levels rose during the Early to Mid-Holocene this land bridge disappeared.

The microlith tradition of Sri Lanka has deep roots that continue through into later periods [36, 59]. The early inhabitants of Sri Lanka occupied areas in and around caves and rock shelters (see sites like Batadomba-lena, Fa Hien-lena and Kitulgala Beli-lena), and had a forest-resource subsistence base [35, 36, 60].

By 12kya, we see some changes in the land use practices of Sri Lanka inhabitants. Sites are found not only in the Wet Zone but also at the interface of the Wet Zone and the Dry Zone, for example Bellan-bandi Palassa, where data suggests that more mixed forest and open conditions may have been exploited alongside the rainforests of the wetter areas [36, 118–120].

A broadening of diet is implied from multiple sites [36]. Some consistency with earlier periods is seen: within the Wet Zone primates make up 70–80% of the mammalian assemblages at these sites over the period of occupation [60]. Beyond primate exploitation, mouse deer, giant squirrel, mongoose, jungle cat, civet were also being utilized [59, 60]. These have limited seasonal fluctuations, and suggest stability in gathering and hunting activities. During the terminal Pleistocene however, an increase in molluscs and small semi-arboreal mammals is suggested by the increase in osseous points relative to microliths at Batadomba-lena [60], and the addition of nuts such as *Canarium* sp. and tubers suggests that processed plant foods may have become a staple part of the diet. The position of sites on the borders of the Wet and Dry Zones would also allow for a range of faunal exploitation, not just semi-arboreal mammals but fully arboreal primates, small ground-dwelling mammals and larger suids. In addition long distance connections are seen, with marine shells found at Batadomba-lena and shark teeth at Bellan-bandi Palassa [60].

While this implies a shift at 12kya to a broader diet and associated lifeway, there has been some question as to how extensively humans were occupying different habitats of Sri Lanka across the Pleistocene-Holocene transition [36]. The mollusc and faunal evidence seems to indicate a variety of paleoenvironments were exploited at 12kya compared with earlier periods, but little research into preservation pathways has been done. How far these data indicate changing local conditions or transport remains to be assessed [36], and as a result Premathilake and Risberg [120] and Boivin et al. [121] have questioned whether humans were indeed solely occupying the tropical rainforests in earlier time periods, or carrying out more mobile subsistence strategies. Beyond these subtle debates however, at 12kya we can say that all lowland occupied parts of Sri Lanka were classified as LU1 HGFF in the 12kya time window.

This brings us to the highlands, and specifically the Horton Plains. The Horton Plains are the central highlands of Sri Lanka, today characterized by mires, plains, forested and grassy hills. The major rivers of Sri Lanka, including the Mahaweli, Kelani and Walawe, all have tributaries that originate in the Plains. Premathilake [119] has argued that there is evidence for oats and barley being cultivated at 11kya based on pollen data. In addition he note the presence of wild rice using bulliform phytoliths at 10kya. The taxonomic use of bulliforms for rice domesticated status has been questioned by scholars such as Pearsall et al. [122] as bulliforms change not due to domestication but due to ecological factors. However the presence of rice along with cereal pollen remains an important factor to note in this region. There is an increasing level of microcharcoal from 13kya also noted [119, 120]. Combined with the pollen assemblage [119], this has been interpreted as showing an increase in forest clearance activities, grazing/pasturing, the possible presence of cultivated plants, disturbances such as opening of grasslands and anthropogenic erosion [119]. The multi-proxy evidence of phytoliths, stable carbon isotopes, organic carbon, microcharcoal and pollen have been argued by [119, 120] to show incipient plant management based around cereals, aligned with the idea of a predomestication cultivation [119, 120]. On this basis, we added to the LU1 HGFF designation, a sub-classification of LU2 low-level food production noted for the upland Horton Plains.

Overall then in the 12kya time window we see a landscape dominated by HGFF land-uses, with small areas of minimal/extensive, and some patches of no human land use. The HGFF land use has some diversity within it, but the coarse nature of the available data makes this difficult to fully assess. Little has changed since the pre-12kya datasets beyond a reduction in data availability. We can contrast with the data from 6kya.

### 4.3 South Asia at 6kya (time window 6,250–5,750 years ago)

Significantly more archaeological sites are known from the 6kya timeslice than in 12kya, though as noted, we face significant difficulties with chronology. By 6kya, new chronological indicators such as ceramics and metal artifacts reduce our reliance on lithic technology as a time marker, though some difficulties remain.

**Fig 5:**
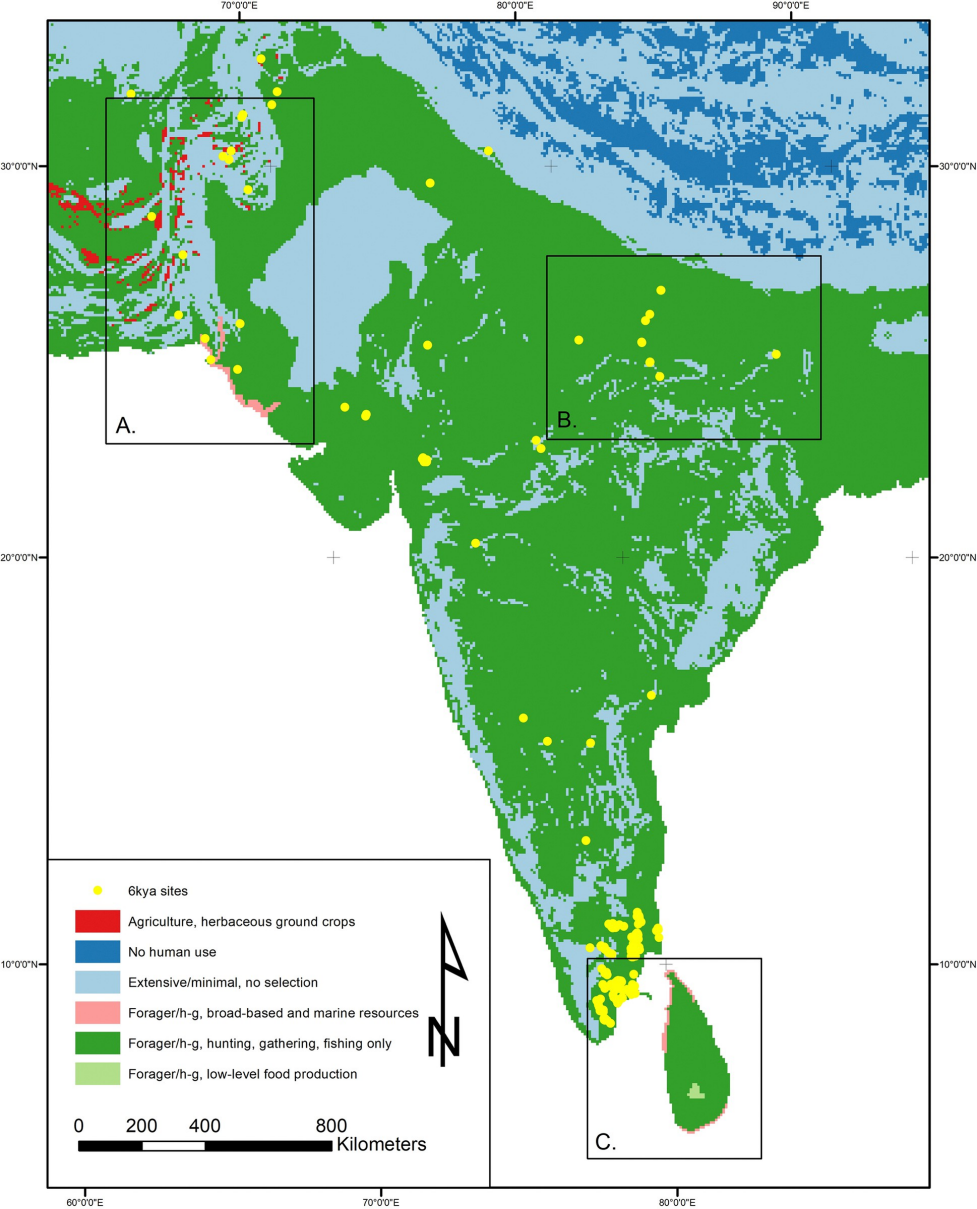
Land use at 6kya (6,250–5,750 years ago). LU1 HHGF remains the dominant land use across the subcontinent, with a slight decrease in minimal-extensive and some increase in LU2 categories. Diversity in the forms of land use therefore seem to increase by 6k, but HGFF remains the main land use across South Asia. It is also important to note the change in sea level to those similar to today, changing the available land from that in 12k to that recognizable today. Map made by authors using ArcGIS and LC6k database.

Interestingly, many (but by no means all) sites with documented 6kya occupation levels continued to be used into later periods. This new pattern is worth noting, suggesting that in some places new patterns of mobility and/or site continuity had begun to be established. While there are some notable patterns of change within the 6kya data, in aggregate, the main pattern is one of continuity. Established 12kya land use practices continued in most places. Although land used for HGFF is still dominant, we can better identify variability within this broad category, and we also see early agriculture emerging as a mosaic of diverse practices, crops, and environmental contexts. Foreshadowing changes seen in later periods (4kya and beyond), in this period we see shifts in forest utilization, intensification in the use of coastal resources, and the start of, or spread of, agriculture (unevenly) across the subcontinent. In this period, the diversity of land use practices and the multiplicity of land use trajectories characteristic of South Asian history is clear (Fig 5).

#### 4.3.1 Early agriculture

A key shift in land use between 12kya and 6kya was the development of agriculture. By the latter period, parts of the Indo-Iranian borderlands and areas around the edges of the Indus floodplain were bring farmed, while on the Gangetic plain, parts of Sri Lanka, and possibly elsewhere, low-level food production coexisted with foraging. While agriculture in the Indo-Iranian borderlands relied on introduced sets of practices and taxa, in other regions we see local developments reliant on native taxa.

In the Indo-Iranian borderlands, there are many sites with evidence of a shift from foraging to the full suite of domestic production, including introduced species like sheep, goat, wheat and barley and the domestication of local species like cattle [123–125]. This includes sites like Mehrgarh, a large archaeological complex along the Bolan River in Baluchistan [126–136], often cited as the earliest evidence for agriculture in the sub-continent [126, 127] due to the discovery of non-native domesticates. In Period I, the 7th millennium BCE (possibly the 8th millennium BCE though this is disputed–see [134] for summary), charred macrobotanical remains provide evidence for a Near Eastern cropping assemblage, dominated by barley [126], along with domesticated einkorn, domesticated emmer, and free-threshing wheat [126, 137]. Period IIA potentially falls in the 6kya time window (though dating is debated–see [134]. Here we see continuity of the earlier Period I patterns: barley continued to be the dominant plant species at the site and buildings interpreted as ‘granaries’ or storage space were also identified [137]. This was accompanied by a local domestication of cattle [125, 138].

Other sites in the Indo-Iranian borderlands include Shei Khan Teraki [139], and Sarai Khola. These last two have both wheat and barley in their Neolithic levels (4th-3rd millennium BCE) [139–142], and new analysis is showing that at Sarai Khola in particular in the earliest levels (Period I 4th-3rd millennium BCE) there was predominantly 6-row barley with small quantities of free threshing wheat but also present were small proportions of local millets (Bates et al. forthcoming). Nineteen other sites are argued by Possehl [143] to be ‘agro-pastoral economies’ which in our classification are termed as LU1 agriculture, with animal domesticates as variables (following [14], while Kili Gul Mohammed has been suggested, based on sheep, goat and oxen remains, to represent a transhumant pastoralist settlement linked to Mehrgarh [129, 134, 139, 141, 143–145]. Overall then this region might have LU1 pastoralism added to the LU1 agriculture. However most of these sites lack archaeobotanical data to allow us to understand what people were doing with plants as well as animals (e.g.: using wild gathered resources or farmed crops) and as such we opted for now to classify these sites as LU1 agriculture, so as not to be biased towards the easier preserved and recovered zooarchaeological data.

**Fig 6:**
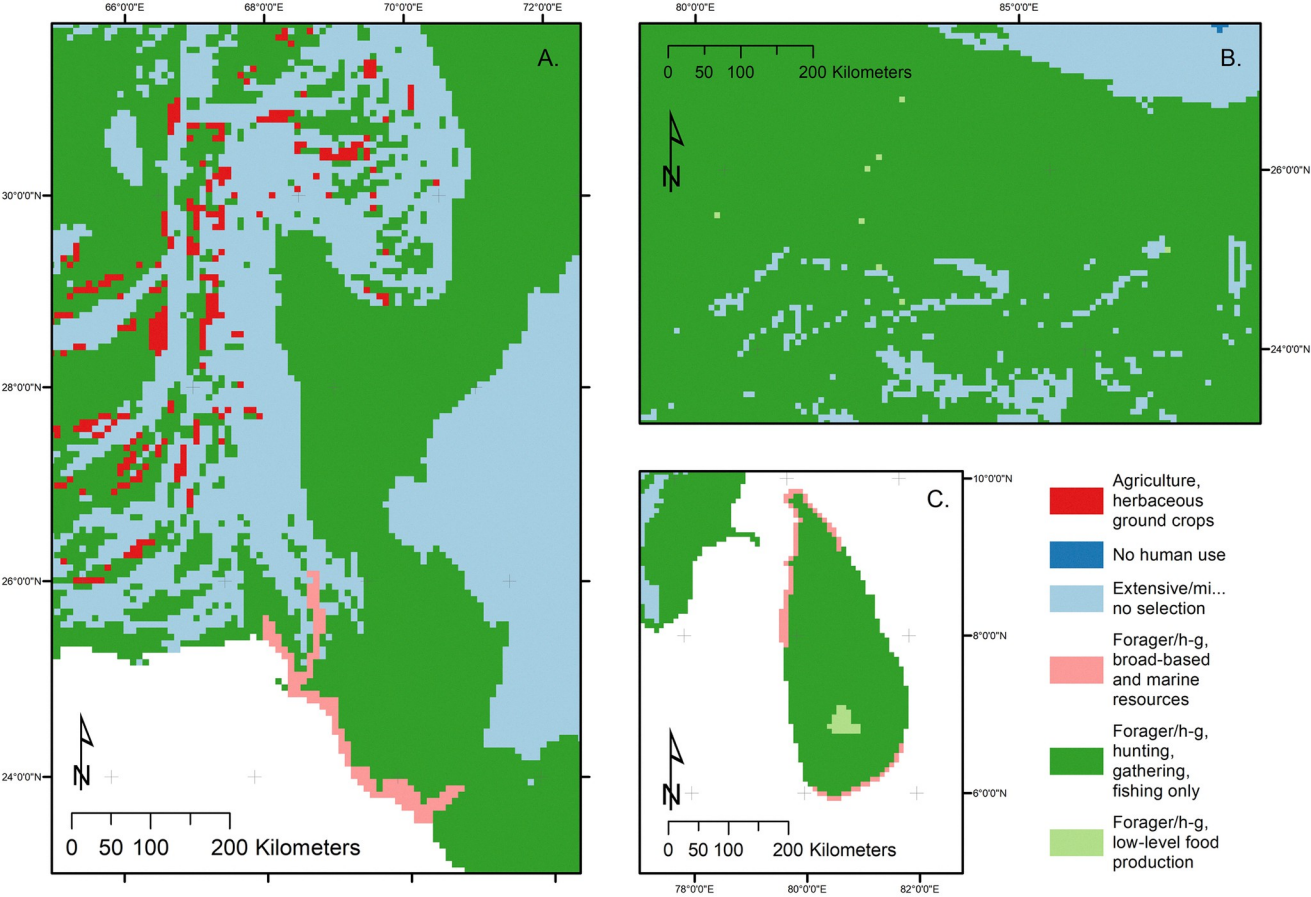
Details of Land Use at 6kya. A) Excerpt map for Indo-Iranian borderlands and Gujarat, showing the diversity of land use within this north west region of South Asian the 6kya time window (6,750–5,5750 years ago). LU1 agriculture and LU2 herbaceous ground crops can be seen in the alluvial fans in this zoomed in excerpt, as can the broad-based marine/aquatic resources along the Gujarat coast, along with areas of LU1 agriculture. B) Excerpt map for Ganges Plains at 6kya. Areas of LU1 HGFF LU2 LLFP can be seen in this zoomed-in map. Map made by authors using ArcGIS and LC6k database. C) Excerpt map of Sri Lanka at 6kya, showing the prevalence of LU2 broad based and/or marine/aquatic resources along the coastlines. The continued presence of LU1 HGFF LU2 LLFP in the Horton Plains is also highlighted. Maps made by authors using ArcGIS and LC6k database.

As in many other regions of the Old World, e.g. the Fertile Crescent, southeast Iran, North China [146–148] early agricultural settlements in extreme northwest South Asia appear to be located not on river plains but on alluvial fans in the hilly flanks [141]. This pattern is related to the critical resource of water in the Indo-Iranian borderlands [149], with alluvial fans presenting small pockets of alleviated conditions [125, 141, 150]. Sites such as Mehrgarh, Kili Gul Mohammed, Sarai Khola and Sheri Khan Teraki are located on alluvial fans—indeed, Petrie and Thomas [141] even suggest that fans should be targeted as locations for future surveys of sites dating from the 6th-4th millennium BC. Based on this pattern, we limited LU1 classifications of agriculture in this region to alluvial fans [141] (Fig 6). To map this, rules for coding the Himalayas and Southern India were combined to identify LU1-“minimal-extensive” areas. Elevations below 4000m, but above ruggedness threshold of 13 were coded as LU1-“minimal-extensive”. Following this, any cells where known sites were identified (e.g.: Mehrgarh) were manually classified as LU1-“agriculture” and LU2-“herbaceous ground crops”, with variables recorded as sheep, goat, cattle, wheat, and barley, then using the pattern suggested by Petrie and Thomas [141] alluvial fans were identified. This was done using the global 280m resolution landform classification dataset [151] and an arbitrary elevation threshold of 500m, and these cells were coded LU1 agriculture LU2-“herbaceous ground crops”. Farming in these semi-arid regions is probably best classified as LU2 herbaceous ground crops, in common with other regions using Near Eastern crops, but as data on fallowing and land use intensity are lacking which would have allowed for a more refined LU categorization [14], this cannot yet be verified. While this has been done in the mapping, it remains speculative, based on the hypothesis of Petrie and Thomas [141], and like all mapping carried out here needs archaeological survey to assess and ground truth the modeling further.

To the east/southeast of the uplifted Indo-Iranian borderlands lies the periodically inundated Indus River flood plain, as well as other flood plains in the Indus River system. This region, known for the later Indus or Harappan Civilization (c. 3200–1500 BCE), poses challenges to archaeological research. As in the 12kya time window, many 6kya sites rest under several meters of alluvial deposits and the current high-water table. However at the edge of the plain, both in the west and in the east handmade pottery with very coarse temper made of plant material (sometime with seed and chaff impressions) has been recovered from surface scatters (Abro and Chandio pers. comm. from unpublished material). Such pottery suggests the presence of groups of early agriculturalists and keepers of domestic animals who might have exploited the Indus plain for both small-scale cultivation and for raising animals. Furthermore, new work in the plains of north Sindh on settlements with a later chronology of Early Harappan occupation shows a very important presence of various species of millet (contrary to a hypothesized Near Eastern package), possibly suggesting early local experimentations in cultivation, and eventually domestication, by foragers/pastoral groups (Madella & Jiménez, pers. comm.).

There is more evidence for early farming settlements outside the alluvial zone. In Haryana, settlements such as Bhiranna have levels dating to the pre-Harappan that fall within the 6kya time window contain ceramics and copper artifacts, and agriculture was the primary subsistence strategy [152–154]. These sites are not numerous and so we have been cautious in coding LU1 agriculture squares in this region based only on sites where they explicitly state agricultural data. In practices this ends up with only Bhiranna coded as LU1 agriculture at 6kya.

As another example of caution in using LU1 agriculture, in North Gujarat taphonomic factors have led to a paucity of plant remains that makes it difficult to trace with sufficient certainty the local trajectories of domestication. However, plant and animal remains from sites such as Loteshwar and Vaharvo Timbo show the presence of both wild and domesticated forms (e.g. *Bos* sp. and *Sesamum* sp.) in the chronology, suggesting at the very minimum that the mid-Holocene groups in the area were experimenting with diverse approaches to food procurement and that eventually human populations adopted a strategy that involved semi-nomadic lifeways with the cultivation of fast-maturing crops (mostly local millets), the gathering of wild plants and the possibility of local animal domestication [155]. In North Gujarat again a cautious approach has been taken and LU1 HGFF with LU2 LLFP was coded to reflect this, again in squares where there were sites with this evidence.

Other sites are also discussed as ‘agricultural’ and deserve a specific discussion in this synthesis. These are the sites in the Gangetic plains, with a special mention of Lahuradewa. Occupation at Lahuradewa dates to the 7th millennium BCE [156–162], with possible evidence of incipient rice agriculture dating to the same time and possibly as far back as the 8th or 9th millennium BCE [159]. However these very early dates have been debated because they are based not on the rice (or any other crop) grains themselves but on bulk charcoal samples, while the rice and other crop remains collected during excavation from the same cultural Period IA show dates of between 6442-2879cal BCE–see Tables 1 and 2 in [159] (see also critiques in 11,153). By the 5th millennium BCE however (and thus potentially intersecting the 6kya time window), rice was an established part of the diet across much of the Gangetic Plain [159, 160, 163, 164].

Debate however centers around whether the rice used at these sites was domesticated or not, and thus whether to classify them as agricultural, low-level food producing (pre-domestication cultivation), or hunting-gathering-fishing-foraging. Tewari et al. [159, 160] have argued that the grains found at Lahuradewa resemble a mix of domesticated and wild types based on morphometrics, and that the four spikelet bases found in Period IA were of the domesticated variety. By Period IB they argue grains and spikelet bases are all domesticated in type. On the other hand, reassessment of morphometric data carried out by Fuller [165] questions this suggesting that there is overlap between domesticated, wild and immature rice, and that the Lahuradewa grains fall closer to the wild types, and that the spikelet bases appear in images to show more immature or wild features than domesticated. More broadly, it has been shown that morphometrics for identification of rice domestication based on grains alone are extremely complex [166]. Rice grain size is linked less with domestication and more with environmental variability, such that small niche changes can lead to significant variations in grain width to length ratios, skewing discrimination analysis on wild versus domesticated status. The spikelet base data is less contentious (outside of Period IA where small sample numbers hamper discussion), but even within Period IB there is still debate over whether these represent a domesticated crop [159] or the product of a green harvest of wild/semi-domesticated rice [167].

As such, although by Lahuradewa Period 1B (within the 6kya time window), rice was an important food resource in the Gangetic plain, it is more plausible it was a gathered wild resource or harvested as pre-domestication cultivation and therefore the square where Lahuradewa is located has been coded in the database as LU1 HGFF and LU2 low-level food production. This fits well with other data from Lahuradewa, such as the faunal assemblage which is made up exclusively of wild species [17] and with the intermittent or seasonal occupation at the site.

Much in the same way that many sites in the Indo-Iranian borderlands have not been the subject of as much analysis compared with the ‘type site’ or Mehrgarh, there are numerous other early settlements in the Gangetic plains that provide evidence for early plant cultivation beyond Lahuradewa. There are several other sites within the 6kya time window with evidence for rice use, but these have less secure dates and lack evidence for detailed spikelet base analysis or grain morphometrics than Lahuradewa. These include Damdama [168], Chopani Mando, Jhusi and Hetapatti [169] in the Ganges plains. At Chopani Mando, wild rice grain impressions in pottery as well as wild animal remains have been reported [89]. It is the presence of rice, and the potential reliance on rice, that makes these sites stand out in comparison with other ‘Mesolithic’ Gangetic sites arguably part of HGFF land use systems. Beyond the rice debate however they are similar in faunal remains and where in the landscape they are located. The querns, rubbers, anvils, hammerstones, and sharpeners of grey sandstone that occur at excavated sites in the region [170], suggest food processing, including, possibly, wild cereal processing [81] like those at Lahuradewa (and those in North Gujarat by way of regional contrast).

The developments seen within the 6kya time window set up many of the developments and debates seen later in 4kya in the Ganges at sites like Chirand [171, 172], Senuwar [173, 174], Magahara [175], Chechar-Kutubpur [176], Sakas [177] and many more sites besides that show continuity in food gathering, hunting and cultivation practices as part of this broader Gangetic Neolithic system that develops from the 5^th^ (possibly as early as 7^th^ millennium BCE) and continues through to the early centuries BCE.

Low-level food production sites in the Ganges are found in association with lakes, along rivulets or on river banks [170, 178]. Watery locations provided ideal contexts for the exploitation of wild Indian rice, that grew in these seasonally monsoon-inundated ponds, via developing cultivation systems. However these spaces were also places where HGFFs continue to occupy for their additional rich resources. This makes coding a specific rule for LLFP difficult, and as such only sites that have data that suggests LLFP explicitly have been coded as this LU2. The complexity of continuity and change between the HGFF patterns we see from 12kya and development of LLFP between 12kya and 6kya make for important targets for future research within a geographically delimited area (see Fig 6).

#### 4.3.2 Continuation of HGFF across the subcontinent

While there are important patterns of change at 6kya with the adoption of agriculture and animal husbandry, land use continuity is the more dominant pattern across the many ecologies and regions of South Asia. For example, in the Indo-Iranian borderlands outside the alluvial fans we see HGFF continuing through and down into the low-mid elevations. Across the subcontinent we see the majority of sites found are labelled as ‘Mesolithic’, an ambiguous category as noted above and often used as shorthand for HGFF. We can use the Ganges as an example of the patterns we have seen in the continuity of HGFF land use across 12kya and 6kya [80, 179–182]. Arguments have been made for environmental and population pressures leading to this spread in Mesolithic tradition [81], but as discussed already, settlements in the Ganges Plain are generally found in three environmental contexts: on banks of lakes, along rivulets and seasonal streams, and in open wooded areas [81]. Sites like Sarai Nahar Rai [39], Damdama [168, 178], and Mahadaha [183] exemplify this. This is a continuity of 12kya patterns where sites are also found in these localities.

The Ganges Mesolithic settlements are considered sedentary for the most part. Plaster huts with plaster floors and evidence of deep and continuous stratigraphy, heavy querns and other material culture, multi-seasonal faunal remains, and repeated evidence of burials [81]. As in other 6kya sites, microliths played an important role in the technological assemblage at the sites, but so too did bone tools. It is likely the microliths were hafted to create composite tools. Pal [81] suggests that microliths tools were important for a set of very diverse processes (scraping, sawing, cutting, drilling, incising, and grooving) applied to very different materials such as hides, dry hide, meat, plant matter (including grasses and wetland species like reeds), wood, bone and antler. Pal [81] notes that dentition microwear from human remains shows a coarse diet consistent with hunting, foraging and gathering, supporting the multiple uses suggested for the tools [184, 185]. This is important, as plant remains are rare in the Ganges at this time (with the exception of LLFP sites discussed above) apart from a few sites like Damdama [168, 178].

Moving out of the river plains we have other environments where HGFF was likely the dominant LU, but it is more difficult to assess this in some areas. For example, in Gujarat, there are numerous sites that could date to 6,000 years ago, seen for example in surveys by Sankalia [186] around Vasad, Subbarao [187] in the Mahi Valley, by Mehta and Sonawane [188] along the Meshwo River, by Momin [189] in Kheda District, in Panchmahals by Sonawane [190] and in Orsang Valley by Ajithprasad [191–193]. Not all these apply to the 6kya time window given that, as noted above, the ‘Mesolithic’ and ‘microlithic’ are complex terms used broadly for a wide time span. Within the 6kya time window, it can be noted that all the environments occupied at 12kya continued to be occupied. Thomas [31] suggests that these locations would have been chosen for factors such as, “water source, availability of food resources and strategies adopted for their exploitation and other considerations like protection from hostile natural agents.” The peoples of these sites utilized a flake-blade industry, with a greater diversity of tool types than in the earlier time window, but with similarities in form continuing in many of these (e.g.: blades, flakes, scrapers). The range of scrapers, retouched blades and flakes, burins, and borers seen suggest new ways of preparing and processing food items at the sites. Indeed, the data coming from Loteshwar, one of the best-studied sites with HGFF microlithic industry in Gujarat, show evidence for intensive food processing of both plants (the evidence includes some small portable grinding stones) and animals, the selective hunting of antelopes and an important exploitation of fish (both from the Rann and rivers/lakes). In Vaharvo Timbo, another site with microlithic HGFF deposits in North Gujarat, people were hunting (mostly blackbuck) and fishing, but there is some evidence for plant exploitation (for a summary see [155].

In this region, then, there appears to be continuity across our two time windows. Water was an important resource, with sites situated close to water sources, whether rivers, seasonal streams, or the coast, as well as near water-collecting escarpments or small seasonal lakes. Sites are also situated on aeolian features, often stabilized fossil dunes, similar to the situation emerging on the edges of the Thar desert. The amelioration of the arid conditions with the onset of Holocene resulted in the stabilization of these dunes and water collection between the dunes (as highlighted by the formation of clay/silty soils similar to black cotton soils), and thus increased vegetation and suitable land for exploit and use.

#### 4.3.3 Change along coastlines, river deltas and on islands

With the changes in sea level seen with the stabilizing of glaciers and melt water in the mid Holocene, significantly different land uses along coastlines and in river deltas are seen where data is available in the 6kya time window. For Sri Lanka, as an example, Roberts et al. [36] suggests the changes across the Early to Mid Holocene reflect a diversification in land use and subsistence strategies, though this could also be a reflection of the changing sea levels, and thus a sampling bias due to many early sites being underwater (see also debates in [101, 103].

As an example of the changes, we see shell middens indicating the development of a more specialized aquatic resource base along the Indus delta and Makran coastlines, and further inland to the mangrove lagoons of Lake Siranda [97, 194–196]. The mangrove swamps and lagoons of the Arabian coastal zone are an environment that has mostly disappeared today [195]. These environments contained *Terebralia palustris* L. and *Telescopium telescopium* L. mangrove gastropods which form the majority of the shells alongside marine gastropods forming middens seen along the coast. Radiocarbon dating of these shells suggests that exploitation of these environments began in the 7th millennium BCE [194]. By 6kya, there was systematic exploitation of these mangrove regions as part of the foraging economy [195], which was enhanced by the rising sea levels, changes in the South Asian monsoon (following [71], and tectonic shifts that created a particularly rich environment for exploitation (see also [196–198]. This region has been coded as LU1 HGFF with LU2 broad-based and/or marine/aquatic resources (see Fig 6).

At the same time, we can contrast these shifts with other deltas and coastlines where there appears to be little change over the 12kya to 6kya. Land use in the Bengal Basin does not appear to change much. While the coastline shifts, as noted in Heroy et al. [109] and Mukherjee et al. [106], human use of the Ganga-Bramaputra-Meghna flood and delta plain continued to be minimal, while the western uplands continued to have HGFF activity on it similar to that seen around 12kya [107, 110, 111]. Sites like Birbhanpur, Paruldanga [110, 112], and Midnapur [110, 111] continue to be occupied in the western uplands with similar land use strategies as in the 12kya time window.

In Sri Lanka we see these patterns of continuity and change exemplified. At 6kya, the exploitation of the Sri Lankan forested regions continues in much the same vein as in the 12kya time window and as such LU1 remains classified as HGFF, with the exception of the Horton Plains that continue as in 12kya, and the coastlines, as described below. The Sri Lankan microlith tool kit can be contrasted with the broader South Asian tool kit. Lewis et al. [57] suggests that the Sri Lanka microlith assemblage shows much greater consistency in form over the Pleistocene and into the Holocene than other regions. As Roberts et al. [36] have noted “the Sri Lankan Microlithic tradition appears to have been a specific and stable environmental and cultural adaptation”.

Evidence of land use diversification within the HGFF category in Sri Lanka comes from a series of coastal shell midden sites, allowing sub-classification of LU2 broad-based and/or marine/aquatic resources (see Fig 6). The land bridge is submerged by 6kya, making Sri Lanka a true island at this point. Shell middens on the south coast such as Patirajawela Site 50, Henagahapugala Site 57, Arnakallu Site 30, Udamalala, Karagan Lewaya, Hungama Mini-athiliya, and Pallamalala and on the north coast such as Matota have been identified [59, 199, 200]. There remains debate about their chronology and cultural formation [119, 200]. Sites like Pallemalal, for instance, contain large shell-beds, with concentrated pockets of shell that can be as wide as 4km [200]. Though their anthropogenic origin has been questioned [119], finds from the shell beds at sites like Pallemalala and Kalametiya [59] would suggest a human component to their use or even creation. The shells are predominantly of *Pelecypoda* bivalves from the *Mactra* genus [200]. These marine shells were found in association with charred bone fragments and stone implements and burnt patches in the shell midden. Microliths similar to those found inland were also seen. While shells formed the primary material in the middens, additional material suggests that the subsistence base was broad, and not solely focused on one resource [200]. A single line of squares along the north and south coast of Sri Lanka where sites with shell middens are located have been coded LU2 broad based and/or marine/aquatic resources to reflect this.

### 4.4 Challenges in the 12kya and 6kya

**Fig 7:**
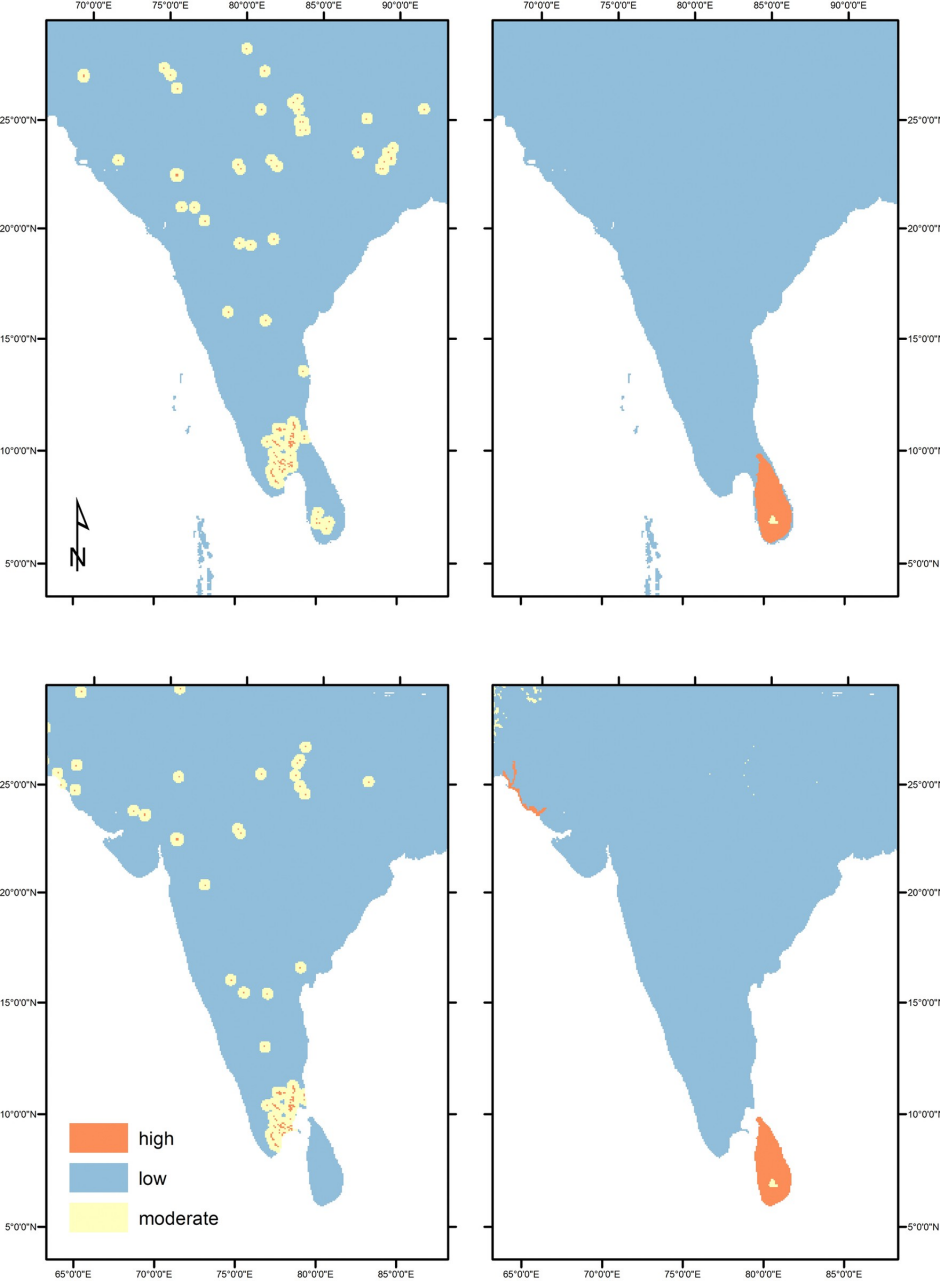
Data coverage and data quality maps. Top left is data coverage at 12kya, top right is data quality at 12kya. Bottom left is data coverage at 6kya, bottom right is data quality at 6kya. Data coverage has been estimated by putting a raster interpolation on site data for both 12kya and 6kya to create density buffers at 30km to take into account survey work around sites. Data quality was mapped using the 8x8km grid square system of the LC6k database. Map made by authors using ArcGIS and LC6k database.

There have been complex challenges at both 12kya and 6kya for modeling LU in South Asia. Data coverage and quality are key among these (Fig 7). The number of sites and radiometric dates are fewer than in many other regions. Data coverage and quality followed appraisal arising from specialist discussion which assessed the reliability of information (the level of quality of available data) and created the rough consensus that allowed us to produce summary coverage maps. Difference of opinion remain, and our database will continue to be updated.

Data coverage refers to the extent to which the land surface has been studied archaeologically while quality is the forms of evidence available [14]. As Morrison et al. [14]: SI) note “Thus, a region with large-scale surface surveys may have good coverage, but in terms of evidence needed to make land use assessments (well-dated, well-located sites with analyzed plant and animal remains, for example), the quality of the data may be low”.

For South Asia at both 12kya and 6kya, both data coverage and quality are often poor. The general pattern in data coverage is mostly low coverage with points of high coverage (sites and well surveyed areas). There is only a slight increase in the number of pockets of variable-high, for example in the Indo-Iranian highlands shell middens indicating the development of a more specialized aquatic resource base at 6kya. Overall then, we see a picture of highly concentrated studies in small regions, with the vast majority of South Asia being understudied during this transition.

Data quality shows a slightly different story. Although data quality for the majority of the sub-continent is low (similar to data coverage) meaning not much material (archaeobotanical, zooarchaeological, dating, geolocated sites etc.) is present for discussion, there are again pockets of variable-high quality information. And this changes between 12kya and 6kya. At 12kya the focus of high-quality data is in Sri Lanka, with the Horton Plains listed as variable due to the debates around dating. Beyond this there are few other points. By 6kya however there is more material for discussion, and these are found at specific sites in the Indo-Iranian foothills, Indus floodplains, Gangetic floodplains, Gujarat and coastal areas where there is an increased focus on how food procurement changes over time. These are however small points in a vast swathe of variable low-quality, as focus remains on single sites rather than larger scale assessment of cultural groups through scientific analyses.

By looking at the data coverage and quality we can highlight areas that need further assessment. While it may seem that the entirety of South Asia at 12kya and 6kya need work to fill this in, we might compare some of the LU mapping with the coverage and quality maps to think about specific regions to target for work. The Western and Eastern Ghats are an example of this. Land use geographical rules had to be extended from the few known sites yet from the coverage and quality maps it is clear that coverage is low to variable at best and quality is low. Much of the work encountered for this region was survey work. Targeted excavations to expand knowledge on the land use are needed here. The same can be said of densely forested regions. These pose a special challenge to archaeological research practically, methodologically and theoretically. While scholars once claimed that autochthonous hunter-gatherers were unable to subsist in tropical forests [201], sufficient evidence to the contrary [202] suggests that poor data coverage and quality may not be entirely a product of historical land use. In the northeastern and eastern parts of the subcontinent, there are some well dated sites in Nagaland, namely Ranyak khen [203–205], Khangkhui Caves [206, 207] and Nongpok Keithelmanbi [208], producing evidence of hunting-gathering groups occupying caves and rock-shelters. Sites in other parts of Nagaland occasionally produce sporadic stone tools, though without much control of stratigraphy and chronology [77]; while suggestive of more extensive land use in this region, they cannot be definitely assigned a LU at 12kya or 6kya with any confidence. Reassessing these with regards to dating, for example, will allow for refinement of both the site and the LU maps at 12kya and 6kya. This kind of targeted work will not only be beneficial for land use mapping as illustrated in this paper but also for the archaeology of 12kya and 6kya South Asia.

## 5. Discussion–continuities and changes in land management across 12kya to 6kya

The synthesis of 12kya and 6kya data across South Asia is the first comprehensive modelling of archaeological data for land use in the sub-continent. Our synthesis, though subject to uncertainties, shows both patterns of continuity and change associated with larger processes of human-environment engagement. Perhaps the most important narrative we can pick out is that while there are some shifts in land use across these time windows, HGFF remained the dominant land use; within this, there was a mosaic of strategies exploiting diverse and complex landscapes and ecologies, a pattern that has continued into the present. While it is not novel to conclude that South Asia is comprised of many niches and a diversity of practices, demonstrating the deep time history of this diversity, including the continued salience of HGFF practices alongside agriculture is an important step for modelling anthropogenic impacts and quantifying the human environmental footprint in the longue-durée. This analysis specifically highlights the role of HGFF, moderating the dominance of the agricultural narrative in South Asian prehistory.

Despite the new development of food production by 6kya by overall area foraging lifeways continued as the dominant land use practice into the 6kya time window. While earlier strategies were also likely to have been diverse, by this period we can better identify how past peoples adapted to the ecological diversity of South Asia. Two important developments are examples of mobility and strategies of regional burning. As noted, coastal shell midden sites suggest patterns of either long-term residential stability and/or structured, recurrent visits to the same location. As agriculture expanded, some hunter-gatherer-fisher-foragers had new resource options, including exchange with agriculturalists or access to domestic animals [22], potentially creating new ways to organize foraging lifeways.

As microcharcoal data increasingly become available from sediment cores [209, 210], we can also begin to track fire histories in South Asia. Both foragers and farmers are known to have practiced landscape-scale anthropogenic burning as part of land management strategies; microcharcoal data suggest that burning increased overall during the mid-Holocene. Data from the salt lakes of the Thar Desert c.6kya suggest that hunter-gatherers around the edges of this arid region were engaged in burning [211–213], while in the Gangetic plains this practice can be pushed back to the 12kya time window but with increasing frequency or intensity by 6kya [157, 158]. At Lahuradewa, microcharcoal data are accompanied by rice bulliform phytoliths from 8kya, which Saxena et al. [214] suggest show cultivated rice, though this is debated (as noted above see [122]) for debates on rice bulliform formation and the use of scallops not as domestication indicators but as environmentally determined). Carbon content peaks have also been noted in the Nilgiris of South India before 3,500 cal BP [215]. As noted, paleoenvironmental evidence from the Horton Plains of Sri Lanka point to a very long history of burning. Ethnographic parallels suggest that anthropogenic burning can aid desired plants and animals [216], and as in other world regions, some historically-known hunter-gatherer in South Asia practiced regular burning, a practice suppressed in the Colonial era [217]. While fire history remains understudied in South Asia, the potentially important role of regional burning underscores the opportunity for hunting and gathering groups–as well as agriculturalists–to affect land cover.

This diversity in HGFF land use is critical to highlight, as it is often masked by discussions of agricultural origins [15–19]. It is important to stress that while there is evidence for agriculture and low-level food production in the Indo-Iranian borderlands, the edges of the Indus plain, and parts of the Gangetic plain, these regions also continued to be used for hunting and gathering. Indeed, at 6kya agriculture and low-level food production occupied only a very small portion of the landscape which continued to be used primarily for foraging. Scholarly interest in agriculture often obscures this important pattern. The presence of farming in a few small areas is easily overblown, especially in analyses using a coarse spatial scale, where these important but small areas of agricultural land use are analytically ‘smeared’ across large areas. Although farming does later become more spatially extensive, at 6kya it was spatially restricted, a pattern obscured by inappropriately teleological analyses.

This is not to say that the development of agriculture and food production was unimportant–it is the beginning of a land use that eventually comes to dominate the sub-continent, but at 6kya was restricted to specific contents.

Our analysis of Early and Mid-Holocene South Asia suggests that the cultural and subsistence diversity of later periods may have a deep history. Thinking through the developments of changing human-plant interactions and associated land use, we see in the examples of the Indo-Iranian borderlands, the Indus plain and the Gangetic plain different approaches to the cultivation of crops. At one extreme, in the Indo-Iranian borderlands we see a focus on an agricultural system of non-native crops while in the Ganges and Gujarat we see the cultivation of native resources as part of HGFF land use systems. In the middle are the Indus plains where we potentially see the beginnings of the exploitation of native resources alongside the addition of some introduced crops. Despite this, in all regions water was a critical factor. In the Indo-Iranian borderlands the shortage of water ensured that only particular landscapes were suitable for agriculture, while in the Gangetic, Gujarati and Indus plains sites may have been chosen to avoid flooding, or to access naturally occurring wild resources. Access to readily available water may have made agriculture/cultivation possible in different areas under different systems–wheat and barley need ready supplies of water but dislike regular flooding conditions that may be hard to control outside intense agriculture settings and so are well suited to the alluvial fans in hilly regions, millets require much less water and are well adapted to hyper arid conditions, while rice is an abundant and accessible resource in water rich environments. Indeed Fuller and Qin [218] have argued that the high productivity and predictability of wild *Oryza* sp. may have meant that it could be effectively exploited in its wild state, discouraging intensive cultivation and thus selective pressures that would eventually lead to domestication. The uneven distribution of research across South Asia suggests that there are many other subsistence regimes yet to be studied; agriculture appears later than 6kya in southern India, for example, and yet we know very little about the land use practices that preceded it.

## 6. Conclusion

The goal of the PAGES LC6k working group has been to reconstruct human land use and land cover on a global scale over the past 12,000 years. Through this, we have sought to understand how humans have transformed the earth across the Holocene. The critical evaluation and improvement of anthropogenic land use and cover change models (ALCC models) used in local and global climate models will allow for a more nuanced understanding of the variable relationships between humans and the world around them over time. As one of the most densely populated parts of the world today, South Asia is a critical location for studies of the effect of land use related land cover change on climate (e.g.: [47]. Intensive agriculture, including large areas of wet rice, forest loss, mining and quarrying, modifications of the water table, and urban expansion have all profoundly affected earth systems. These conditions are historically derived, however, and to date we do not have a complete picture of how land use has changed through time in this important region.

Work is ongoing to improve the LC6k methodology including comparing different mapping methods, utilizing alternative proxies as well as ways of evaluating available proxies, and homogenizing the analytical approaches used throughout the various LU working groups. This is therefore a beginning. However, our analysis of land use at 12kya and 6kya builds a foundation for a more comprehensive analysis of South Asian land use trajectories, with research on land use at 4kya and 2kya in progress. At the same time, this work also creates a dataset for exploration and mapping of the nature of South Asian hunter-gatherer-fisher-forager diversity in deep time, as well as early agricultural developments. Our synthesis makes clear the urgent need for additional research, especially for radiometric dates and analysis of plant and animal remains. As a work in progress, we expect our ability to understand the diversity and complexity of South Asian land use histories to continuously improve, sharpening our understanding of the evolution of human-environment relationships and their import for the present and future.
